# Detailed head localization and incidence of skin cancers

**DOI:** 10.1038/s41598-021-91942-5

**Published:** 2021-06-11

**Authors:** Marta Fijałkowska, Mateusz Koziej, Bogusław Antoszewski

**Affiliations:** 1 Department of Plastic, Reconstructive and Aesthetic Surgery, Second Chair of Surgery Medical, University of Lodz, Lodz, Poland; 2 Department of Anatomy, Jagiellonian University Medical College, Krakow, Poland

**Keywords:** Cancer, Anatomy, Medical research, Oncology

## Abstract

Skin cancers are the most common neoplasms; frequently, they localize on the face. The aim of paper is to present the incidence of skin tumors in a single center from 2017 to 2019, describe trends in its frequency and find relations between neoplasms and sex, type of cancer, and its size. An analysis of histopathological files from the surgical department between 2017 and 2019 was calculated. These items were selected: sex, age, type of skin cancer, subtype of basal cell carcinoma (BCC), grading of squamous cell carcinoma (SCC), localization and dimensions of the tumor. The study sample consisted of 387 cases. BCC was the most common cancer and its nodular type was the most frequent. In older patients, the vertical dimension of excised carcinoma was significantly larger. Moreover, this connection was detected only in women compared to men. There were statistically significant differences between dimensions of the skin cancer and sex. In men group, skin cancers had statistically higher vertical dimensions and larger surface areas. On the face and head, BCC more often localizes in the nasal area, while SCC on the auricle. It has been demonstrated that the older the patient, the larger the vertical dimension of the tumor. As such, tumor size is larger in men than in women, as women usually see their physicians sooner than men: cosmetic concerns are more important to them.

## Introduction

When assessing statistics about the frequency of human cancers, one can read that breast, lung, prostate, and colorectal cancers affect millions of people every year. However, the most common neoplasms in humans are skin cancers: one in every three diagnosed malignancies is skin cancer^1^. The actual frequency is hard to establish, especially when basal cell carcinoma (BCC) is considered. Only a few population-based cancer registries collect BCC data, and they usually publish only the first primary (histologically-confirmed) case per patient, due to practical problems in coding multiple BCCs^2^. Additional issues connected with keratinocyte carcinoma, e.g., BCCs, is that they have a good prognosis with high cure rates if diagnosed early and treated^3^. Despite the type of the skin cancer, its progression can cause significant tissue destruction (penetrating subcutaneous tissue, fascia, muscle, perichondrium or periosteum, and cartilage or bone); some surgical reconstruction would be necessary. Aesthetic aspects of reconstruction should be considered, when cancer is in the facial area. Skin cancers are health issues, with an economic burden on society and health services^4^.

The most common skin tumors are BCC, squamous cell carcinoma (SCC), and malignant melanoma (MM)^1,3,4^. BCC and SCC are known as keratinocyte carcinoma (KC), historically termed non-melanoma skin cancers, which correspond to 95% of malignant skin tumors^3,5^. Although melanoma comprises only 1% of all skin cancers, it accounts for approximately 90% of deaths associated with cutaneous tumors^6–8^. BCC rarely results in death or metastatic disease, but can cause significant morbidity due to destructive local spread^9^. SCC has metastatic potential, with a rate of approximately 1% to 4%, while the risk is higher in organ transplant recipients and immunosuppressed individuals^10^. The general prognosis of metastatic SCC is rather poor^10^. Mortality is primarily associated with regional and nodal metastases vs. distant metastases^10^. Cutaneous melanoma is potentially the most dangerous form of skin tumor, and ranks fifth as the most common in men and seventh in women^11^. Survival rates of advanced melanoma are low; the 5-year survival rate in patients with stage IV melanoma is only about 6%^12^.

The aim of this paper is to present the incidence of skin tumors in a single center from 2017 to 2019, and to describe the trends in frequency and the correlation between neoplasms and sex, type of cancer, and its dimensions.

## Material and methods

This is an epidemiological, descriptive, andretrospective study based onthe analysis of histopathological files from the surgical department in Lodz (Poland). The city is in the center of the country, as well as central Europe, with a temperate climate. Polish people are white European, benefiting as a homogenous group. We collected data from January 2017 to December 2019 (three years). The following items were chosen for further analysis: sex, age, type of skin cancer, subtype of BCC, grading of SCC, anatomical localization of the tumor and its dimensions (vertical-height, horizontal-length, and forward/backward-width) measured by a pathologist during microscope examination. The surface is calculated by approximation, using an equation of an ellipse surface [P = pi*length/2*width/2]. Anatomical body parts affected with cancer are: head (face/scalp), neck, trunk, upper limb, lower limb, and dorsum. The project was approved by ethics review board of Medical University of Lodz (approval no. RNN/364/18/KE), and conducted according to the Declaration of Helsinki. Permissions were collected in the form of written informed consent obtained from all patients.

Collected data were grouped on Microsoft Excel spreadsheets and analyzed with STATISTICA v.13.1 (StatSoft Inc., Tulsa, OK, USA) for Windows. Quantitative features were usually characterized with the mean value ± standard deviation (SD). Median and quartiles were applied if a non-parametric test was performed to compare quantitative features in each group. Qualitative features were presented as frequencies and percentages. The qualitative variables were compared by the χ2 test of proportions for categorical variables (two-sided). The Shapiro–Wilk test determined if the quantitative data were normally distributed. To verify homogeneity of variance, Levene’s test was used. To compare two groups, the Mann–Whitney or t-test was used. Spearman’s correlation coefficient assessed the association between values. Power analysis indicated that to detect a significant correlation, R = 0.20 used a two-sided test, a 5% significance level test (α = 0.05), with 80% power (β = 0.2), with the minimal sample size being approximately 193. A p-value of < 0.05 was considered to be statistically significant.

### Ethical approval

The project was approved by ethics review board of Medical University of Lodz (approval no. RNN/364/18/KE).

## Results

In total, there were 359 patients included in the study: 145 (40.4%) men and 214 (59.6%) women. Fourteen patients (3.9%) had skin cancer excised more than once, thus our study sample consisted of 387 cases. The mean patient age was 71 years (SD ± 11.9 years), the youngest patient was 30, and the oldest was 96. Nearly 90% (88.9%) of patients were 60 or older. There was no difference between sex and age, as the mean age of female patients was 71 years, and that of male patients was 71.1 years. In most cases, the skin cancer was localized to the head (348; 89.9%), with remaining localizations listed in Table 1. The methods of introduced reconstructive procedures were mainly dependent on the size of lesion. After excision of small tumors direct closure was possible in 254 cases. If not, local skin flaps (75 cases) or skin grafts (58 cases) were performed. As most of cancers were localized on the face, local flaps used for reconstruction were mainly random ones. The retroauricular area or the inner surface of the arm area were mainly used as donor side for skin grafts. All surgical procedures were performed according to plastic techniques and standards so functional and esthetic outcomes were satisfactory and highly accepted by patients.

**Table 1:** Study group characteristic.

Variable	N; mean	%; SD
Patients	359	
Men	145	40.4
Women	214	59.6
Cancers	387	
Mean age	71.0	11.9
**Anatomical Location**		
Head	348	89.9
Neck	4	1.0
Trunk	9	2.3
Upper limb	10	2.6
Lower limb	5	1.3
Dorsum	11	2.8

*SD* standard deviation.

Skin tumors were divided into 4 main groups (BCC, SCC, MM, and others), presented in Table 2. The occurrence of BCCs and SCCs were similar across a 3-year interval for the examined population (343, 88.6% and 37, 9.6% respectively). Four cases of melanoma were seen, representing only 1% of the total sample. However, BCC is the most frequent skin cancer followed by SCC and melanoma, this is typical distribution known in literature and it was also confirmed in our research. In addition, there were 3 tumor cases defined as ‘other’: eccrine carcinoma, hidradenocarcinoma, and trichoblastic carcinoma. In further research only BCC and SCC were analyzed.

**Table 2:** Occurrence of most common types of skin cancers across 2017–2019.

Year	n total of cancers	BCC		SCC		Melanoma		Other	
		n	%	n	%	n	%	n	%
2017	125	110	88.0	12	9.6	0	0.0	3	2.4
2018	142	130	91.5	11	7.7	1	0.7	0	0.0
2019	120	103	85.8	14	11.7	3	2.5	0	0.0
Total	387	343	88.6	37	9.6	4	1.0	3	0.8

*BCC* basal cell carcinoma, *SCC* squamous cell carcinoma.

Cases of BCC were divided into subtypes, with the nodular type most common (229; 66.8% of total BCC), followed by ulcerative (10.5%), infiltrative (8.5%), and superficial (7.3%). Basosquamous and cystic types were the least frequent in the current study (16 cases, 4.7% and 8 cases, 2.4%, respectively). Values of surface area and vertical dimensions of the cancer can be seen in Table 3. SCC has a significantly larger surface area when excised (SCC: 0.5 [0.31; 1.02] vs. BCC: 0.38 [0.2; 0.79], *P* = 0.042), and were less superficial than BCC (SCC: 0.30 [0.2; 0.5] vs. BCC: 0.2 [0.1; 0.3], *P* = 0.009).

**Table 3:** Size parameters of keratinocyte carcinoma, data presented as average or median.

Cancer	Specific	n	%	Vertical dimension (mm)	Surface (mm^2^)
BCC	Total	343		0.23 ± 0.16	0.38 (0.20; 0.79)
	Nodular	229	66.7	0.23 ± 0.17	0.34 (0.20; 0.78)
	Ulcerative	36	10.5	0.27 ± 0.15	0.51 (0.24; 1.38)
	Infiltrative	29	8.5	0.19 ± 0.11	0.40 (0.20; 0.79)
	Superficial	25	7.3	0.21 ± 0.17	0.59 (0.37; 1.82)
	Basosquamous	16	4.7%	0.39 ± 0.28	0.30 (0.20; 0.50)
	Cystic	8	2.3%	0.23 ± 0.06	0.40 (0.22; 1.18)
SCC	Total	37		0.39 ± 0.29	0.50 (0.31; 1.0)
	In situ	6	16.2%	-	0.51 (0.2; 1.0)
	G1	21	56.8%	0.43 ± 0.26	0.56 (0.37; 1.9)
	G2	9	24.3%	0.2 (0.1; 0.3)	0.50 (0.24; 1.3)
	G3	1	2.7%	-	-

*BCC* basal cell carcinoma, *SCC* squamous cell carcinoma.

BCC and SCC were found in a facial location (Table 4). Analysis revealed that orbital (28.2%) and nasal (26.7%) areas were most common on the face. SCC is significantly more frequent in the auricular area than BCC (*P* < 0.001). All cases of auricular SCC developed in males, while BCC was associated with the nasal area (*P* = 0.001), and has no sex preference.

**Table 4:** Occurrence of keratinocyte carcinoma depending face area.

Skin cancer	Frontal		Auricular*		Nasal*		Orbital		Perioral		Cheek		Scalp		Tempora	
	n	%	n	%	n	%	n	%	n	%	n	%	n	%	n	%
**BCC (n = 309)**	32	10.4	6	1.9	91	29.4	90	29.1	7	2.3	48	15.5	11	3.6	24	7.8
Basosquamous	2	13.3	0	0.0	6	40.0	4	26.7	0	0.0	0	0.0	1	6.7	2	13.3
Cystic	0	0.0	0	0.0	0	0.0	3	37.5	0	0.0	3	37.5	2	25.0	0	0.0
Ulcerative	4	13.3	1	3.3	8	26.7	4	13.3	1	3.3	7	23.3	1	3.3	4	13.3
Infiltrative	2	7.7	1	3.8	13	50.0	2	7.7	0	0.0	7	26.9	0	0.0	1	3.8
Nodular	23	10.6	3	1.4	59	27.3	75	34.7	6	2.8	29	13.4	6	2.8	15	6.9
Superficial	1	7.1	1	7.1	5	35.7	2	14.3	0	0.0	2	14.3	1	7.1	2	14.3
**SCC (n = 35)**	2	5.7	10	28.6	1	2.9	7	20.0	2	5.7	8	22.9	2	5.7	3	8.6
In situ	0	0.0	2	50.0	0	0.0	0	0.0	0	0.0	1	25.0	0	0.0	1	25.0
G1	2	9.5	5	23.8	1	4.8	4	19.0	2	9.5	5	23.8	1	4.8	1	4.8
G2	0	0.0	3	33.3	0	0.0	2	22.2	0	0.0	2	22.2	1	11.1	1	11.1
G3	0	0.0	0	0.0	0	0.0	1	100.0	0	0.0	0	0.0	0	0.0	0	0.0
Total	34	9.9	16	4.7	92	26.7	97	28.2	9	2.6	56	16.3	13	3.8	27	7.8

*BCC* basal cell carcinoma, *SCC* squamous cell carcinoma.

*SCC significantly more frequent affect auricular area than BCC (*P* < 0.001). In the other hand, BCC were more associated with nasal area than SCC (*P* = 0.001).

**Figure 1:**
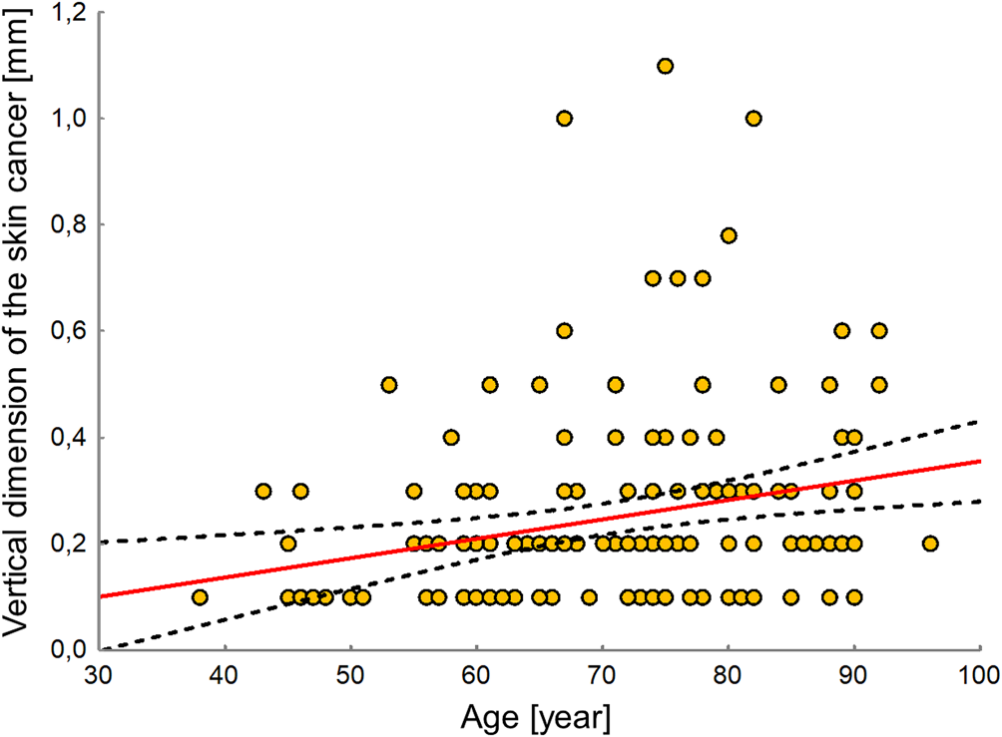
The association between age and skin cancer’s vertical dimension.

It was shown that age was significantly correlated to the vertical dimension of the cancer (R = 0.25, *P* < 0.002; Fig. 1); however, it was not associated with the cancer surface area (*P* = 0.19), or its dimensions (*P* = 0.23–0.25). In older patients, the vertical dimension of excised carcinoma was significantly larger. Moreover, this connection was detected only in women (R = 0.30, *P* < 0.007) compared to men (*P* = 0.29).

**Figure 2:**
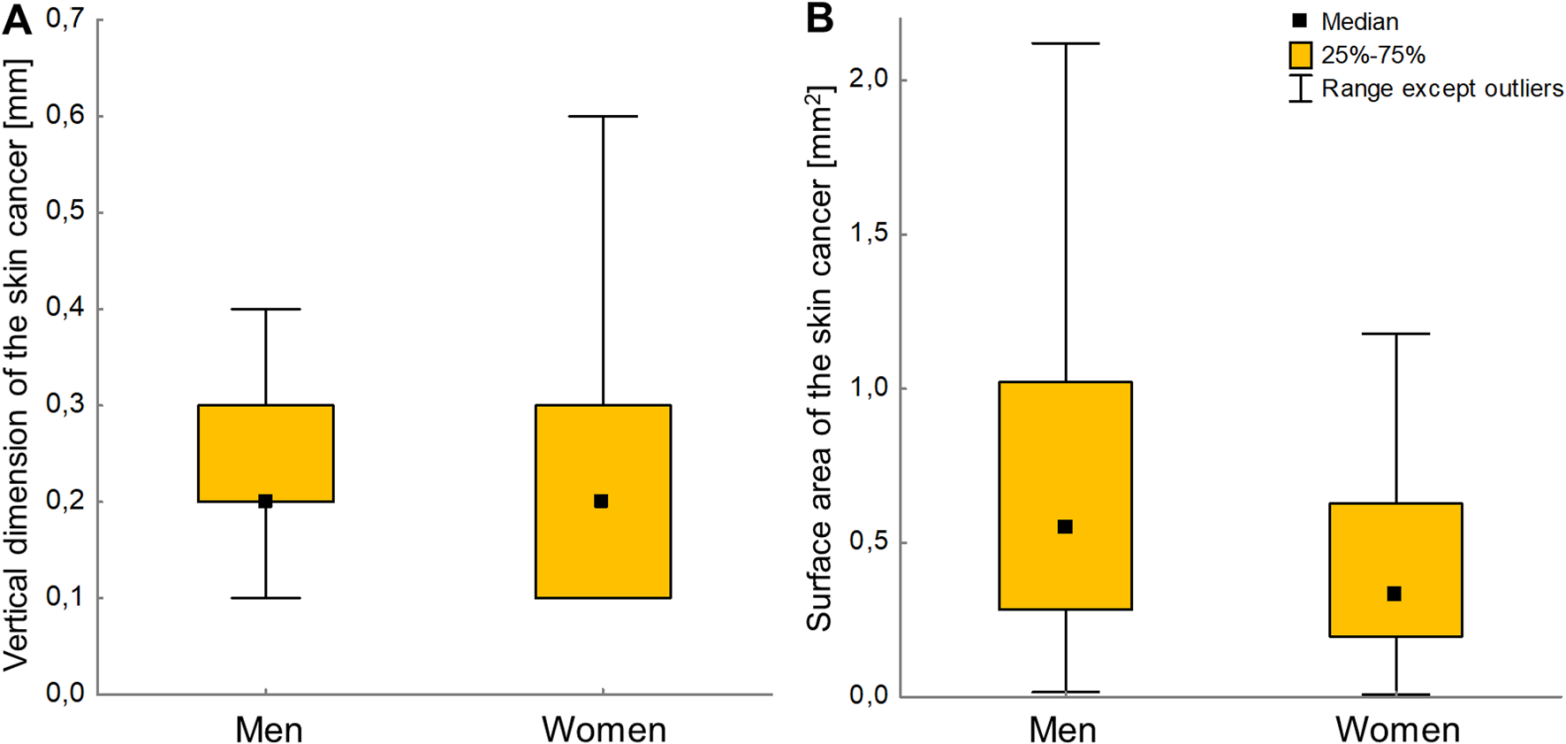
Comparison of skin cancer’s vertical dimension (**A**) and its surface area (**B**), as observed between sexes.

There were statistically significant differences between vertical dimensions of skin cancer, as well as surface area, between sexes. In men group, skin cancers had statistically higher vertical dimensions (M: 0.2 [0.2; 0.3] vs. W: 0.2 [0.1; 0.3]; *P* = 0.01) and larger surface areas (M: 0.5 [0.3; 1.0] vs. W: 0.3 [0.2; 0.6]; *P* < 0.01) (Fig. 2).

## Discussion

According to the World Health Organization (WHO), between two to three million KCs and 132,000 MMs are diagnosed annually worldwide, with increasing frequency^13^. There are many articles in the literature presenting the incidence and distribution of skin cancers from different global centers. Such studies are able to make comparisons between different populations, as it is well-known that environmental factors play a role in the etiopathogenesis of skin tumors^14^.

From 2004 to 2006, Seretis et al. studied skin tumors in the Greek population. The authors analyzed 179 cases of KCs, most including BCC or SCC. BCC was localized on the nose and cheek, while SCC was on the lower lip and auricle^14^. In this population, most patients were over 60 years of age (90% BCC and 83% SCC)^15^. Rezende et al. conducted similar research on a Brazilian population from 2013 to 2015; they analyzed 306 cases of all skin cancers, mostly BCCs, followed by SCCs, and MMs^3^, localized to the face, while MMs were most common on the trunk. In addition, most patients were 80 years or more^3^. We presented data for over 3 years (2017 to 2019), and collected 387 cases of skin tumors. In all studies, BCC was most common, followed by SCC and MM. One similarity is that skin tumors frequently affected older people, with nearly 90% of our patients over 60 years of age. Highly characteristic is that skin neoplasms appear on the face, according to Seretis et al. BCC is typically on the nose and SCC is on the ear, also observed in our study: this could be related to the fact that both Polish and Greek individuals are white European.

One risk factor in skin cancers is chronic ultraviolet (UV) radiation exposure^14^. Such lesions usually appear on body areas exposed to the sun (head and neck, trunk, arms and legs)^14^. Rezende et al. described the distribution of skin tumors as being in both photo-exposed areas and non-photo-exposed areas^3^. Yet, the authors noted that most cases of skin cancers appeared on photo-exposed areas (85.7% BCC and 67.9% SCC)^3^. In our sample, skin tumors affected the head (face and scalp 89.9%), followed by the back (2.8%) and upper limbs (2.6%). On the face, common areas were the nose (BCC) and auricle (SCC), as they are prominent parts of our faces. All cases of auricular SCC affected men, probably due to their short hair, seen in most male patients. In these situations, the ears are not covered, and are frequently exposed to sun. Scalp skin in older, balding men does not tend to be protected by a cap. Results show that scalp cancer comprises 5.7% of SCC cases and 3.6% of BCC cases.

Recently it was described that chronically sun-exposed skin amongst of white Northern European present two types of ageing: hypertrophic photoaging and atrophic photoaging^16,17^. Hypertrophic photoaging is characterized by deep, coarse wrinkles, homogeneity of skin color and few skin cancers; while atrophic photoaging is described with fine wrinkling, a shiny appearance, erythema, telangiectasia, dyspigmentation and a tendency to develop invasive skin cancers^16,17^. What is more interesting atrophic photoaging is more common in males while hypertrophic photoaging in females^18^. This can be explain by the fact that men have different behavior pattern with respect to sun exposure when compared to women^18^. Male patients demonstrate more outdoor work and hobbies than females and what is the most important they show a high reluctance to use sunscreen^18^. It is also perfectly seen in our study because the prevalence of auricle and scalp cancers is higher in men that women with male refusal to use sun blocker, especially on this skin areas which are the most exposed to sun.

In the study based on large sample (ca 13,000 participants) conducted by Ciążyńska et al., the incidence of BCC and SCC were reported^19^. All major areas on the body were taken into consideration. It has been reported that nodular subtype was the most common type of BCC, followed by the superfcial and infltrative subtypes. In current study similarly nodular subtype was the most frequent, however, second place was assigned to ulcaretive subtype followed by infiltrative and superficial. Moreover, they have observed that the superfcial BCC subtype was more common on photoprotected areas, as well as the nodular BCC subtype occurred on the face.

Khalesi et al. presented a meta-analysis to evaluate epidemiologic relationship between pigmentary characteristics and risk of BCC^20^. The authors found that the risk of occurrence of BCC was strongly associated with the following features: red hair, fair skin color, and having skin that burns and never tans. All other factors (like eye color; skin phototype; the presence of melanocytic nevi) had weaker but also positive associations with BCC^20^.

This study has some limitations: using one center for the research, and it was retrospective in nature. It is well-known that patients with skin cancers can be treated in other departments as well, such as dermatology or oncology, where e.g., radiotherapy can be introduced^21^. Hence, our department is surgical one in cases of skin tumors we are suggesting surgical removal of lesion. When patient is afraid of operation or in elderly patients we suggest to perform consultation in oncology department to verify if radiotherapy is applicable in their case. The amount of such patients is not big in our hospital, it is about 5%. Additionally, we are the only plastic surgery department in our region, with most skin tumors affecting the face, and patients preferring treatment with a plastic surgeon. We can assume that these cases will be managed at our center. Our sample was a convenience sample, so selection bias cannot be excluded. Our research describes the correlation between cancer, sex, and age, which is statistically significant. To our knowledge, other studies with detailed analyses regarding localization on the head and dimensions were not previously conducted.

## Conclusions

On the head, basal cell carcinoma often appears in the nasal area, while squamous cell carcinoma appears on the auricular area. It was statistically demonstrated that the older the patient, the larger the vertical dimension of the tumor. The size of the tumor is larger in men than women, which results from women seeing their physicians sooner than men do, even if for aesthetic reasons. From clinical point of view the size of excised tissues will be higher in men that in women, so in men population the methods of reconstruction should be more advanced. Due to the higher vertical dimension of tumor in elderly the thickness of excised tumor should be sufficient enough to perform complete excision of cancer. We recommend that all doctors attend to their male patients, examining their skin and informing them about the need to remove any suspicious lesions.
